# Avian Reovirus σB Interacts with Caveolin-1 in Lipid Rafts during Dynamin-Dependent Caveolae-Mediated Endocytosis

**DOI:** 10.3390/v14102201

**Published:** 2022-10-06

**Authors:** Yuyang Wang, Yangyang Zhang, Wei Zuo, Zongyi Bo, Chengcheng Zhang, Xiaorong Zhang, Yantao Wu

**Affiliations:** 1 Jiangsu Co-Innovation Center for Prevention and Control of Important Animal Infectious Diseases and Zoonoses, College of Veterinary Medicine, Yangzhou University, Yangzhou 225009, China; 2 Testing Center, Yangzhou University, Yangzhou 225009, China; 3 Joint International Research Laboratory of Agriculture and Agri-Product Safety, The Ministry of Education of China, Yangzhou University, Yangzhou 225009, China

**Keywords:** avian reovirus, σB, caveolin-1, lipid rafts, caveolae, endocytosis

## Abstract

Caveolin-1 (Cav-1) is the basic component of caveolae, a specialized form of lipid raft that plays an essential role in endocytic viral entry. However, the evidence of direct involvement of caveolae and Cav-1 in avian reovirus (ARV) entry remains insufficient. In this study, the membrane lipid rafts were isolated as detergent-resistant microdomains (DRMs) by sucrose gradient centrifugation, and the capsid protein σB of ARV was found to associate with Cav-1 in DRMs fractions. Additionally, the interaction between ARV σB protein and Cav-1 was demonstrated by immunofluorescence co-localization and co-immunoprecipitation assays. Furthermore, we found that the internalization of ARV is sensitive to caveolae and dynamin inhibitors, while it is insensitive to clathrin inhibitors. In conclusion, these results indicate that the ARV σB protein interacts with Cav-1 during dynamin-dependent caveolae-mediated endocytosis for the entry of ARV.

## 1. Introduction

Avian reovirus (ARV) is an important pathogen that can cause severe diseases in domestic fowl, leading to considerable losses [1,2]. ARV is a member of the Orthoreovirus genus of the Reoviridae family [3]. Avian reovirions are non-enveloped icosahedral particles containing a genome of 10 double-stranded RNA (dsRNA) segments encased in two concentric protein shells with spikes projecting from the inner core to the outer layer [4]. These dsRNA segments encode eight structural proteins (λA, λB, λC, μA, μB, σA, σB, and σC) and four nonstructural proteins (μNS, σNS, p10, and p17) [5].

Virus entry into cells is one of the most critical stages of the infectious cycle, and it depends on the viral usurpation of normal cellular processes [6]. Viruses can be taken into cells in two major ways: membrane fusion and the endocytic pathway [7]. Specifically, the major endocytic pathways utilized by viruses are clathrin-mediated endocytosis and caveola/raft-mediated endocytosis [8,9]. Lipid rafts are dynamic membrane microdomains enriched in cholesterol sphingolipids and specific associated proteins that function in various biological processes [10]. Caveolae are a subset of lipid rafts, and the essential structural component is caveolin-1. Caveolae participate in endocytosis, vesicular trafficking, cholesterol transport, and cell signaling [11,12]. 

In recent years, increasing evidence has indicated that uptake of some viruses occurs via the caveola/raft-mediated endocytic pathway, although most viruses are taken into the host cells by clathrin-mediated endocytosis [9,13,14,15,16,17]. Since the lipid bilayers of enveloped viruses are from the host plasma membrane, the majority of studies focus on the role of rafts in the entry and egress of enveloped viruses [9]. By comparison, the function of lipid rafts in the replication of non-enveloped viruses is just beginning to be explored, such as SV40, Human adenoviruses (HAdVs), and Echovirus 1 [18,19]. Recently, a few reports have described how lipid rafts are required for ARV and Muscovy duck reovirus (MDRV) entry into host cells. Using chemical inhibitors, siRNA, and fluorescence imaging, Huang et al. suggested that ARV might enter cells through a caveolin-1-mediated and dynamin-2-dependent endocytic pathway [20]. Wu et al. evaluated the effects of raft-disrupting agents on the replication of MDRV and found that MDRV enters susceptible cells via a caveolae-mediated endocytosis-like pathway [21]. Our previous study also indicated that cellular cholesterol in lipid rafts plays a critical role in ARV replication through the depletion of cholesterol by methyl-β-cyclodextrin (MβCD), cholesterol replenishment, and microscopy-based co-localization analysis [22].

Nevertheless, there is still a lack of evidence to prove that lipid rafts/caveolae are directly involved in ARV entry, and more research is needed to draw conclusions. Furthermore, relevant studies have indicated that ARV entry is more complicated than previously perceived. For example, it has been reported that one reovirus can hijack multiple endocytic pathways for cell entry, and reoviruses change entry pathways in different cell lines. Moreover, even different rotavirus strains can enter the same host cell through different endocytic pathways [23,24,25,26]. Thus, the precise mechanism utilized by ARV to enter cells requires further characterization. ARV σB is the major outer capsid protein, which appears to be a good candidate for involvement in early virus–host interactions in ARV infection [4,27]. Although σB is likely to be responsible for early virus–host interactions, there is little research on the role of σB in ARV infection. In this study, Vero and DF-1 cells were chosen as models to clarify the entry routes of ARV strain GX/2010/1. Membrane lipid rafts were isolated as DRMs by sucrose gradient centrifugation after cells were infected with ARV. We found ARV capsid protein σB appeared in Cav-1 rich fraction in DRMs, indicating that the ARV σB protein is probably localized in lipid rafts/caveolae. In addition, confocal microscopy and co-immunoprecipitation analyses were carried out, and we demonstrated for the first time that the ARV σB protein is associated with Cav-1 in cells. Dynasore is a specific dynamin inhibitor [28,29]. Chlorpromazine (CPZ) is used to block clathrin lattice dissociation and disrupt clathrin-mediated endocytosis [30], and Nystatin is a pharmacological blocker of caveolae-mediated endocytosis [31]. These specific chemical inhibitors were used to investigate the dependence of ARV strain GX/2010/1 entry into Vero and DF-1 cells on dynamin, caveolar, and clathrin. The inhibitor studies validated the role of caveolae in ARV endocytosis. This study may help to clarify the entry routes of ARV and understand the virus–host interactions during the ARV internalization. 

## 2. Materials and Methods

### 2.1. Cells and Virus 

Vero and DF-1 cells were maintained in Dulbecco’s modified Eagle’s medium (DMEM; Gibco, Shanghai, China), supplemented with 10% fetal bovine serum (FBS) (Gibco) at 37 °C in a 5% CO_2_ incubator. The ARV strain GX/2010/1 used in this study has been described previously [32,33].

### 2.2. Reagents and Antibodies 

Chlorpromazine hydrochloride (CPZ) and Dynasore were purchased from Absin Bioscience Inc. (Beijing, China). Nystatin and 4′,6-diamidino-2-phenylindole (DAPI) were purchased from Sigma-Aldrich (Beijing, China). Rabbit monoclonal antibodies against Cav-1 and Myc-Tag, and a mouse monoclonal antibody against Flag-Tag were purchased from Cell Signaling Technology (Danvers, MA, USA). Mouse polyclonal antibodies against σB and μNS of ARV were prepared in our laboratory. A mouse monoclonal antibody against GAPDH and secondary antibodies, including fluorescein isothiocyanate (FITC)-conjugated goat anti-mouse IgG, rhodamine isothiocyanate (TRITC)-conjugated goat anti-rabbit IgG, and horseradish peroxidase (HRP)-conjugated goat anti-mouse/rabbit IgG, were purchased from Thermo Fisher Scientific (Shanghai, China). RIPA lysis buffer, PMSF, and SDS-PAGE loading buffer, anti-Flag/Myc, and mouse IgG magnetic beads were purchased from Beyotime Biotechnology (Shanghai, China).

### 2.3. Construction of Expression Vectors

The ARV strain GX/2010/1 S3 (GenBank accession No. KJ476707) was amplified by PCR and cloned into a pCDNA-3.1(+) vector (Invitrogen, Waltham, MA, USA) to generate plasmids pCDNA-Flag-σB. The forward primer was 5′- AGCGGTACCATGGAGGTACGTGTGCCAAACTTTC-3′ and the reverse primer was 5′-CGCCTCGAGTTACTTATCGTCGTCATCCTTGTAATCCCAACCACACTTCACAACAG-3′. The primers and the Cav-1 gene (GenBank accession No. NM_001105664) were synthesized by GenScript (Nanjing) Co., Ltd. (Nanjing, China). The Cav-1 gene was cloned into the pCDNA-3.1(+) vector (Invitrogen) to generate pCDNA-Myc-Cav-1. 

### 2.4. Detergent-Resistant Membranes (DRMs) Extraction by Density Gradient Centrifugation

Vero and DF-1 cells were infected with ARV at a multiplicity of infection (MOI) of 50 for 1 h at 37 °C; the ARV-infected cells grown in monolayers in 100-mm culture dishes were collected and the DRM fractions were isolated from cells as previously described [34]. The cells were washed thrice with ice-cold phosphate-buffered saline (PBS) and collected in 2-mL complete cell lysis TNE buffer (25 mM Tris-HCl [pH 7.5], 150 mM NaCl, and 5 mM EDTA) containing 1% Triton X-100 (Sigma-Aldrich, Shanghai, China) and 1% protease inhibitor (Beyotime Biotechnology, Shanghai, China). The lysates were then passed through a tight-fitting glass Dounce Homogenizer 20 times. Subsequently, the lysates were brought to a concentration of 45% sucrose by adding 2 mL of 90% sucrose in TNE buffer and placed at the bottom of a 13-mL ultracentrifuge tube. Thereafter, 4 mL each of 35% and 5% sucrose in TNE buffer was overlaid by gently adding the solutions down the side of the tube. Samples were centrifuged by a gradient at 200,000× *g* for 18 h in a SW41Ti rotor (Beckman Coulter, Brea, CA, USA) at 4 °C. Twelve 1-mL fractions were collected from the top to the bottom of the gradient. Each fraction was resolved by SDS-PAGE and analyzed using Western blotting.

### 2.5. Plasmid Transfection

Transfection was performed using Lipofectamine 3000 (Invitrogen), according to the manufacturer’s instructions. Briefly, Lipofectamine 3000 (5 μL in 6-well plates), the plasmid (5 μg in 6-well plates), and P3000TM (10 µL in 6-well plates) were diluted in Opti-MEM. The diluted DNA was added to diluted Lipofectamine 3000 (1:1 ratio), incubated for 15 min, and then added to the cell cultures. The cells were incubated for an additional 36 h before being assayed.

### 2.6. Indirect Immunofluorescence Assay (IFA) and Confocal Microscopy

IFA combined with confocal microscopy was used to evaluate the subcellular localization of σB protein and Cav-1. Endogenous Cav-1 was detected by rabbit monoclonal anti-Cav-1 antibody; the overexpressing Myc-tagged Cav-1 and Flag-tagged σB protein were detected by rabbit monoclonal anti-Myc and mouse monoclonal anti-Flag antibodies, respectively. Briefly, cell monolayers on coverslips were fixed with 4% formaldehyde for 20 min, permeabilized with 0.1% Triton X-100 in PBS for 5 min, and blocked with 5% BSA in PBS for 45 min. For each group, the cell monolayer was treated with the respective primary antibodies at a dilution of 1:200 in 1% BSA in PBS for 12 h at 4 °C and incubated with the secondary antibodies (FITC-conjugated goat anti-mouse IgG and TRITC-conjugated goat anti-rabbit IgG) at a dilution of 1:500 for 1 h at room temperature. The nuclei were stained with DAPI, and multichannel images of the samples were acquired using an LSM 880 NLO laser scanning microscope and ZEN 3.0 software (Carl Zeiss AG, Jena, Germany).

### 2.7. Co-Immunoprecipitation (Co-IP) Assay

The expression vectors pCDNA-Flag-σB and pCDNA-Myc-Cav-1 were co-transfected into Vero and DF-1 cells. Then, 36 h post-transfection, the cells were washed with cold PBS and lysed with RIPA lysis buffer containing 1% PMSF at 4 °C for 15 min. The cell lysate was centrifuged at 12,000 rpm for 5 min at 4 °C to retain the clarified lysate. Anti-Flag/Myc magnetic beads precleaned with TBS (20 mM Tris, 0.137 M NaCl, pH 7.6) were added to the clarified lysate and mixed at 4 °C for overnight incubation. Thereafter, the magnetic bead mixture was placed on a magnetic separator and separated for 10 s to remove the clarified lysate. After washing with TBS thrice, the magnetic beads were re-suspended, mixed with the Flag/Myc peptide eluent, and incubated for 2 h at 4 °C. The eluent was collected by separating the magnetic bead mixture using magnetic separation and then analyzed by SDS-PAGE and Western blotting. The total cell lysates were used as input samples, and the lysates incubated with mouse normal IgG magnetic beads were used as control.

### 2.8. Cell Infection and Drug Treatments

Specific inhibitory drugs were used to validate the role of caveolae in ARV endocytosis and investigate the entry routes of ARV. Vero and DF-1 cells were grown in monolayers in 12-well plates. The cells were incubated with inhibitors prior to ARV exposure. Briefly, the cells were treated with the indicated concentrations of dynasore (25, 50, and 100 µM), nystatin (10, 20, and 30 µM), and CPZ (5, 10, and 15 µM) for 1 h at 37 °C. The medium containing the drugs was removed, and the cells were washed thrice with PBS. After infection with ARV at an MOI of 10 for 1 h at 37 °C, the virus-containing medium was removed, and the cells were treated with citrate buffer (40 mM citric acid, 10 mM KCl, 135 mM NaCl, pH 3.0) for 40 s to inactivate bound but unpenetrated virions. After washing, fresh medium supplemented with 2% FBS was added. The cells were further incubated at 37 °C, and ARV internalization was evaluated by qRT-PCR and Western blotting at 9 h post-inoculation (hpi). Moreover, since ARV induces extensive syncytium formation in Vero and DF-1 cells, a syncytium formation assay was used to evaluate the level of ARV infection. The cells in each group were fixed with methanol and Giemsa stained at 12 hpi. Multinuclear cells enclosed within a single plasma membrane (number of nuclei ≥3) were considered syncytia. The number of syncytia in three random fields using a ×10 objective was counted. The data are means ± SD of per microscopic field from three independent experiments [35].

### 2.9. Real-Time Quantitative Reverse Transcription-PCR (qRT-PCR)

To monitor the ARV load following drug treatment, qRT-PCR was used to quantify the mRNA levels of the μNS and σC genes. The primers for μNS, σC, and the reference β-actin gene used in real-time PCR are listed in Table 1. Cells were lysed using a TRIzol reagent RNA kit (CWbio, Beijing, China), and total RNA was isolated according to the manufacturer’s instructions. The PCR conditions were 50 °C for 2 min, 95 °C for 2 min; 40 cycles of 95 °C for 15 s, 60 °C for 15 s, and 72 °C for 1 min; the melting curve was determined in three steps: 95 ºC for 15 s, 60 °C for 1 min, and 95 °C for 15 s. The target genes were amplified in triplicate using PowerUp™ SYBR™ Green Master Mix (Thermo Fisher Scientific-Applied Biosystems, Shanghai, China). The data were analyzed using the comparative threshold cycle (CT) method. The housekeeping β-actin gene was used as a reference control.

### 2.10. SDS-PAGE and Western Blot Analyses

SDS-PAGE and Western blot analyses were performed as previously described [36]. Equal amounts of protein were loaded into each sample. After transfer and blocking, the membranes were incubated with the respective primary antibodies at room temperature for 4 h (mouse polyclonal anti-σB, mouse polyclonal anti-μNS, rabbit monoclonal anti-Cav-1, rabbit monoclonal anti-Myc, mouse monoclonal anti-Flag, or mouse monoclonal anti-GAPDH antibodies). Next, the membranes were washed several times and incubated with the appropriate horseradish peroxidase-conjugated secondary anti-mouse antibodies for 1 h. Finally, the blots were developed using an enhanced chemiluminescence detection system (Tanon Imager, Shanghai, China). The level of respective protein was quantitated by densitometric analysis using ImageJ software. GAPDH was included as an internal control. The relative levels of μNS protein in comparison with ARV group that without drug treatment were analyzed, and the ratios are displayed as fold changes.

### 2.11. Statistical Analysis

All statistical tests were performed using GraphPad Prism 8.0 (GraphPad Software, San Diego, CA, USA). The data are expressed as the mean ± standard deviation. Significance was determined by one-way ANOVA (*, *p* < 0.05; **, *p* < 0.01).

## 3. Results

### 3.1. ARV Capsid Protein σB Presented in Cav-1 Rich Fraction in DRMs

**Figure 1:**
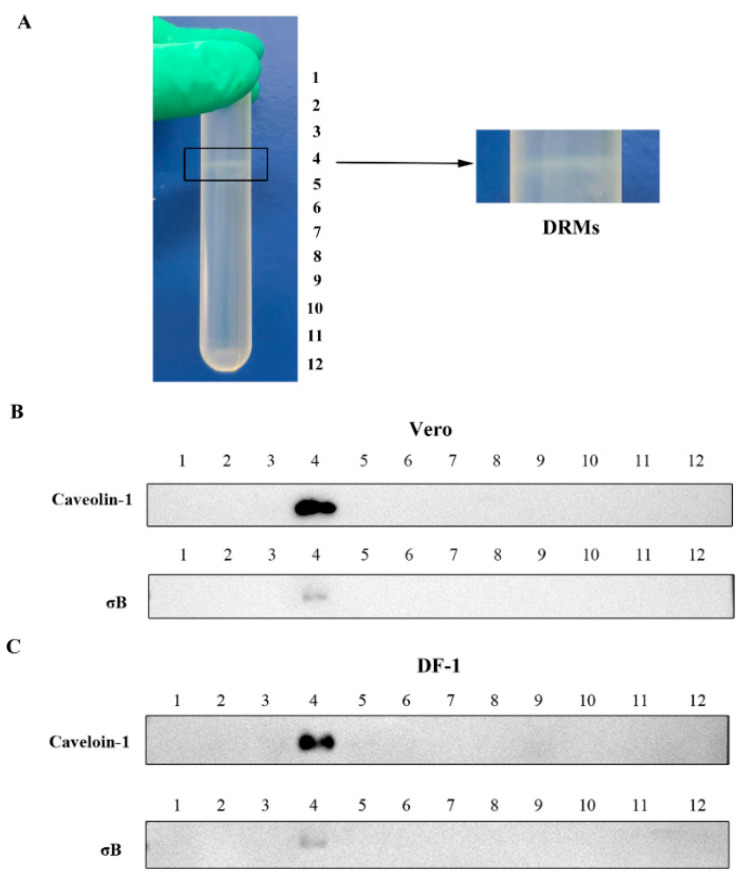
ARV σB protein is associated with Cav-1 in DRMs fractions. (**A**) Detergent-resistant membranes (DRMs) extraction by density gradient centrifugation float to the 35 and 5 % interface. (**B**,**C**) The DRMs fractions were isolated from Vero (**B**) and DF-1 (**C**) cells infected with ARV at 1 hpi. The cells were lysed with cold TNE buffer containing 1% Triton X-100 and extracted via density gradient fractionation. Twelve 1-mL fractions were collected from the top to the bottom of the gradient, defined as fractions 1 to 12, respectively. Each fraction was analyzed by SDS-PAGE and Western blotting, using polyclonal anti-σB and monoclonal anti-Cav-1 antibodies.

To determine whether the ARV σB is associated with Cav-1 in the early stage of ARV infection, caveolae in lipid rafts were isolated from ARV-infected cells 1 hpi. Triton X-100 extraction at 4 °C with discontinuous sucrose gradient centrifugation is the most commonly used method for lipid raft isolation. Using this approach, lipid rafts can be isolated from cellular membranes as DRMs, which are present in the light density fractions as microsome-like structures. In the present study, the DRMs float to the 35 and 5 % interface (fraction 4 and 5 in theory) after ultracentrifugation (Figure 1A). All 12 fractions from ultracentrifugation were collected and analyzed by Western blotting using specific antibodies against ARV σB protein and Cav-1 (marker protein of lipid rafts). As shown in Figure 1B,C, Cav-1 protein was detected in fraction 4, which was defined as a DRM fraction. Moreover, ARV σB protein was detected in fraction 4 of both Vero and DF-1 cells, indicating its association with Cav-1 as early as 1 hpi. 

### 3.2. Co-Localization of σB with Cav-1 during ARV Endocytosis

**Figure 2:**
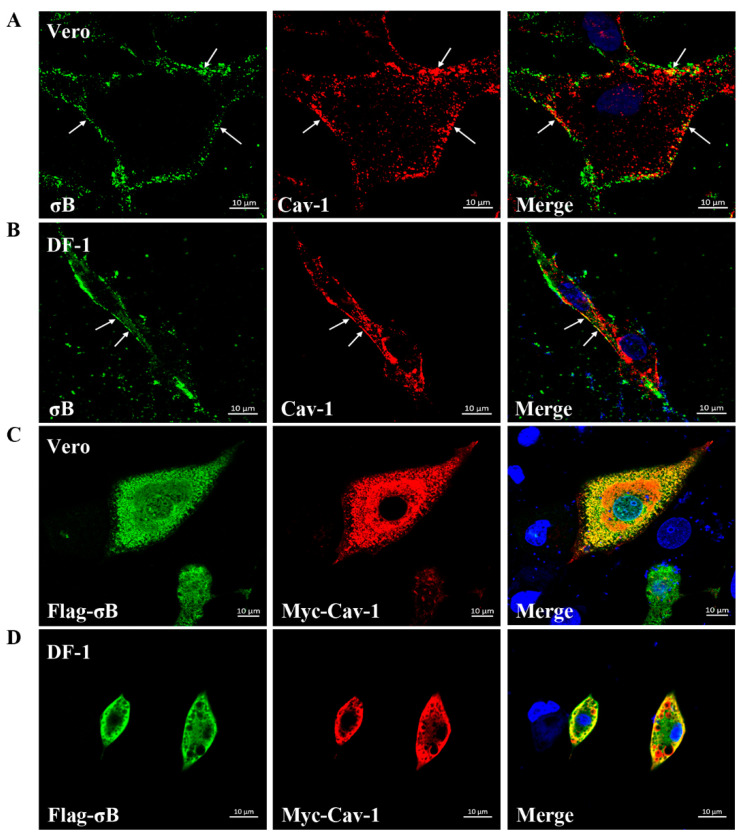
Co-localization of σB with Cav-1. (**A**,**B**) Vero (**A**) and DF-1 (**B**) cells were infected with ARV at an MOI of 10. By immunostaining using antibodies against σB (green) and Cav-1 (red), co-localization of σB with Cav-1 was detected at 40 min post-infection. DAPI was used to stain the nuclei (blue). The white arrows indicate co-localization sites at the cell membrane (yellow). (**C**,**D**) Vero (**C**) and DF-1 (**D**) cells were co-transfected with plasmid pCDNA-Flag-σB and pCDNA-Myc-Cav-1. Immunostaining was conducted using monoclonal antibodies against Flag (green) and Myc (red). DAPI was used to stain the nuclei (blue). A large number of co-localization sites (yellow) of σB with Cav-1 were detected at 36 h post-transfection.

To evaluate whether ARV virions localized in caveolae during virus endocytosis, co-localization analysis of endogenous Cav-1 with outer capsid protein σB of ARV was carried out after cells were infected with ARV at an MOI of 10 for 40 min at 37 °C. As shown in Figure 2A,B, the cells were immunostained with anti-Cav-1 and anti-σB antibodies, and σB protein was found to partially colocalize with endogenous Cav-1 on the cell membrane in both Vero and DF-1 cells. To further investigate the interaction between ARV σB protein and Cav-1, Vero, and DF-1 cells were co-transfected with plasmids pCDNA-Myc-Cav-1 (expressing Myc-tagged Cav-1 protein) and pCDNA-Flag-σB (expressing Flag-tagged σB protein). Cells were fixed and stained with anti-Myc and anti-Flag antibodies at 36 h post-transfection. As expected, a large number of Flag-σB localized to Myc-Cav-1 in both Vero and DF-1 cells (Figure 2C,D).

### 3.3. ARV σB Protein Interacts with Cav-1

**Figure 3:**
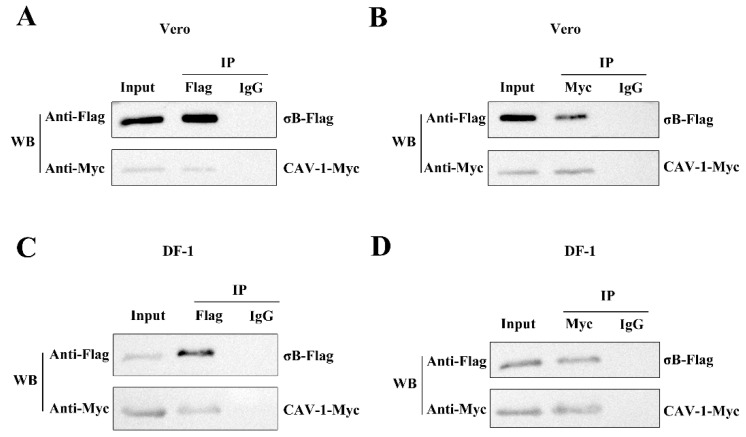
ARV σB protein interacts with Cav-1. (**A**,**B**) Co-IP analysis was performed with Vero cells overexpressing Flag-σB and Myc-Cav-1 at 36 h post transfection. (**A**) Myc-Cav-1 was shown to co-precipitate with the Flag-tagged σB in Vero cells. (**B**) Flag-tagged σB was found to co-precipitate with Myc-tagged Cav-1 in Vero cells. (**C**,**D**) Co-IP assay was performed with DF-1 cells overexpressing Flag-σB and Myc-Cav-1 at 36 h post transfection. (**C**) Myc-Cav-1 was shown to co-precipitate with the Flag-tagged σB in DF-1 cells. (**D**) Flag-tagged σB was found to co-precipitate with Myc-tagged Cav-1 in DF-1 cells.

To further confirm the interaction of ARV σB with Cav-1 in Vero and DF-1 cells, Co-IP assays were performed with cells overexpressing Myc-tagged Cav-1 and Flag-tagged σB protein at 36 h post transfection. As shown in Figure 3A,C, the Vero or DF-1 cells were lysed and immunoprecipitated with anti-Flag or normal IgG magnetic Beads, and elution with 3× Flag peptide. Precipitates and whole cell lysates were probed for Myc-tagged Cav-1 and Flag-tagged σB protein by Western blotting. Myc-Cav-1 was shown to co-precipitate with the Flag-tagged σB. As presented in Figure 3B,D, after incubation with anti-Myc or normal IgG magnetic beads and elution with c-Myc peptide, Flag-tagged σB was also found to co-precipitate with Myc-tagged Cav-1 in both Vero and DF-1 cells. These results suggest that the ARV σB protein interacts with Cav-1 in vitro. 

### 3.4. ARV Are Internalized by Dynamin-Dependent Endocytosis 

**Figure 4:**
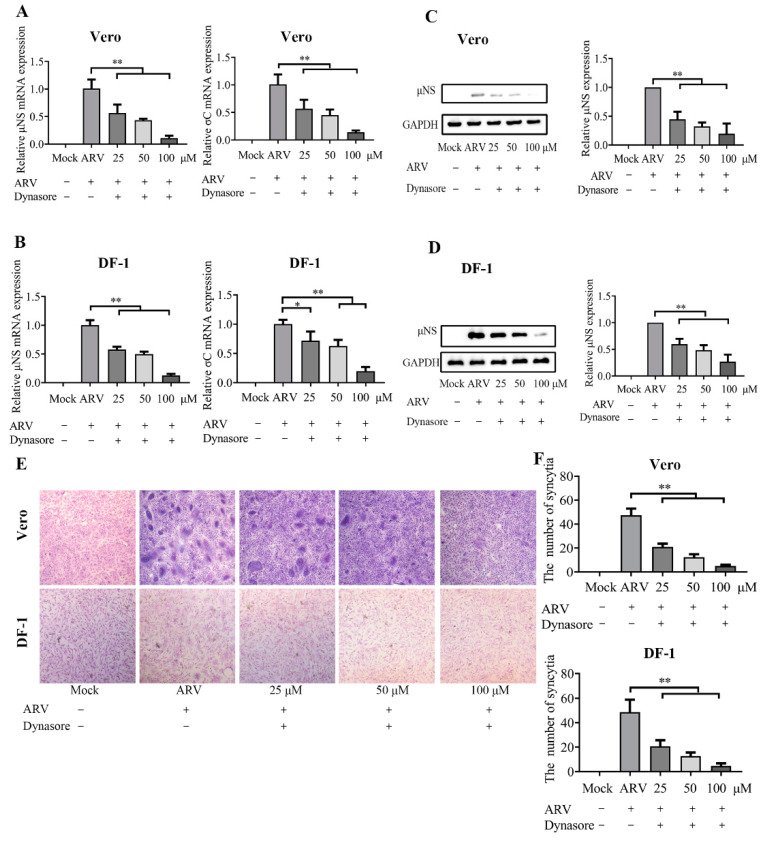
ARV internalization depends on dynamin. Vero and DF-1 cells were pretreated with the indicated concentrations of dynasore (25, 50, 100 μM) for 1 h and incubated with ARV (10 MOI) for 1 h at 37 °C. The bound but unpenetrated virions were inactivated with citrate buffer. (**A**) The mRNA expression levels of the ARV-μNS and σC genes in Vero cells were determined by qRT-PCR at 9 hpi. (**B**) The mRNA expression levels of the ARV-μNS and σC genes in DF-1 cells were determined by qRT-PCR at 9 hpi. (**C**,**D**) The level of ARV μNS protein in Vero (**C**) and DF-1 (**D**) cells was analyzed by Western blotting at 9 hpi. ImageJ software was used to analyze the relative levels of μNS protein in comparison with ARV group that without drug treatment, and the ratios from three independent experiments are displayed as fold changes in the histograms. (**E**) The level of syncytium formation in Vero and DF-1 cells was detected by Giemsa-staining at 12 hpi. Images were captured on an inverted microscope at 10× objective. (**F**) The number of syncytia per microscopic field in Vero and DF-1 cells. Results are presented as the means ± SD of data from three independent experiments. *, *p* < 0.05; **, *p* < 0.01.

The clathrin- and caveolae-mediated endocytosis is the major endocytic pathways utilized by virus entry. Dynamin is critical for both clathrin- and caveolae-mediated endocytosis and functions in detachment-coated vesicles from the plasma membrane. To investigate the dependence of dynamin on ARV entry in Vero and DF-1 cells, different concentrations (25, 50, and 100 μM) of dynasore were used to block coated vesicle formation at viral entry. The mRNA expression levels of the μNS and σC genes are presented in Figure 4A,B, and the levels of μNS protein detected by Western blotting is shown in Figure 4C,D. The results of syncytium analysis in each group are shown in Figure 4E,F. The data suggested that dynasore treatment remarkably inhibited ARV entry in a dose-dependent manner in both Vero and DF-1 cells. The data indicated that ARV enter cells via endocytosis, and dynamin is required for ARV internalization.

### 3.5. The ARV Endocytosis Process Is Clathrin Independent 

**Figure 5:**
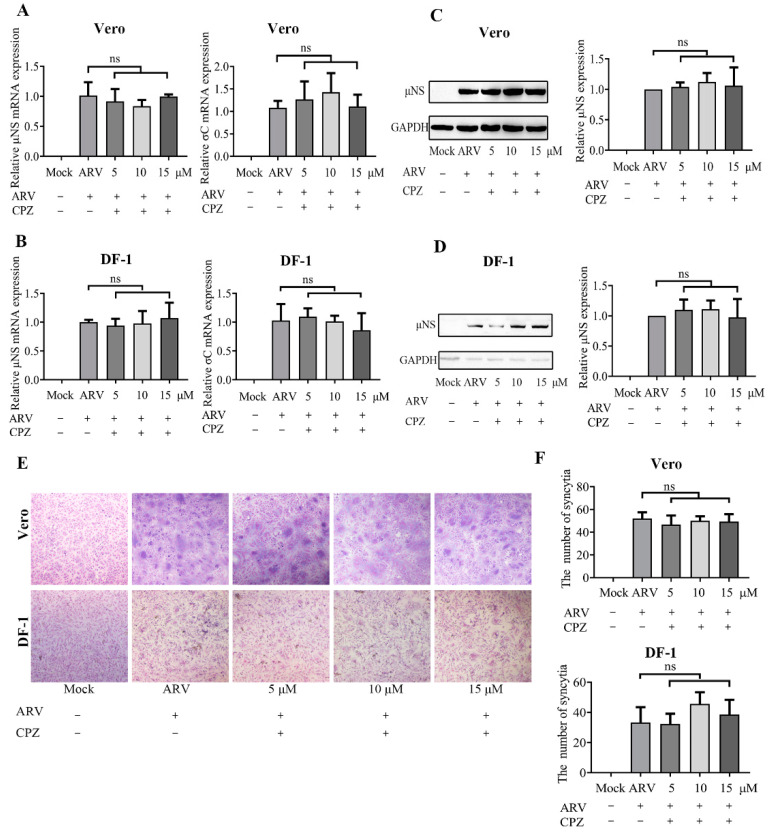
Clathrin is not required for ARV entry. Vero and DF-1 cells were pretreated with the indicated concentrations of CPZ (5, 10, 15 μM) for 1 h and incubated with ARV (10 MOI) for 1 h at 37 °C. The bound but unpenetrated virions were inactivated with citrate buffer. (**A**) The mRNA expression levels of the ARV-μNS and σC genes in Vero cells were determined by qRT-PCR at 9 hpi. (**B**) The mRNA expression levels of the ARV-μNS and σC genes in DF-1 cells were determined by qRT-PCR at 9 hpi. (**C**,**D**) The level of ARV μNS protein in Vero (**C**) and DF-1 (**D**) cells was analyzed by Western blotting at 9 hpi. ImageJ software was used to analyze the relative levels of μNS protein in comparison with ARV group that without drug treatment, and the ratios from three independent experiments are displayed as fold changes in the histograms. (**E**) The level of syncytium formation in Vero and DF-1 cells was detected by Giemsa-staining at 12 hpi. Images were captured on an inverted microscope at 10× objective. (**F**) The number of syncytia per microscopic field in Vero and DF-1 cells. Results are presented as the means ± SD of data from three independent experiments. ns, not significant.

Before ARV infection, Vero and DF-1 cells were pretreated with CPZ at various concentrations (5, 10, and 15 μM). As shown by the viral load, protein levels, and syncytium data in Figure 5, there was no significant difference between the CPZ- and mock-treated ARV infection groups in Vero and DF-1 cells, indicating that the disruption of clathrin-mediated endocytosis had little influence on ARV infection in Vero and DF-1 cells.

### 3.6. ARV Entry Involves Caveolae-Mediated Endocytosis 

**Figure 6:**
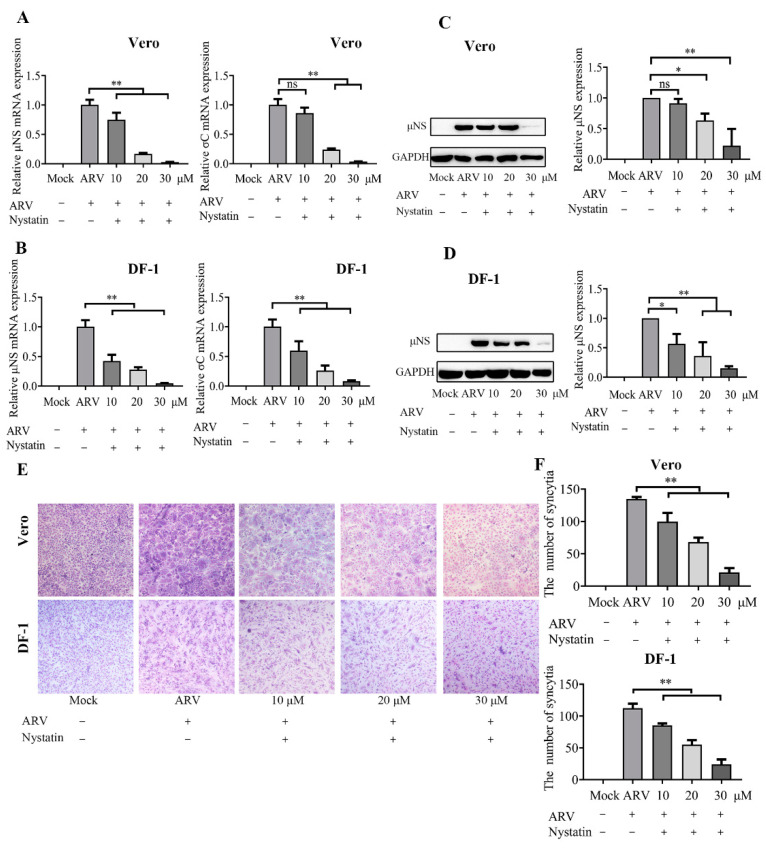
ARV entry is caveola dependent. Vero and DF-1 cells were pretreated with the indicated concentrations of nystatin (10, 20, 30 μM) for 1 h and incubated with ARV (10 MOI) for 1 h at 37 °C. The bound but unpenetrated virions were inactivated with citrate buffer. (**A**) The mRNA expression levels of the ARV-μNS and σC genes in Vero cells were determined by qRT-PCR at 9 hpi. (**B**) The mRNA expression levels of the ARV-μNS and σC genes in DF-1 cells were determined by qRT-PCR at 9 hpi. (**C**,**D**) The level of ARV μNS protein in Vero (**C**) and DF-1 (**D**) cells was analyzed by Western blotting at 9 hpi. ImageJ software was used to analyze the relative levels of μNS protein in comparison with ARV group that without drug treatment, and the ratios from three independent experiments are displayed as fold changes in the histograms. (**E**) The level of syncytium formation in Vero and DF-1 cells was detected by Giemsa-staining at 12 hpi. Images were captured on an inverted microscope at 10× objective. (**F**) The number of syncytia per microscopic field in Vero and DF-1 cells. Results are presented as the means ± SD of data from three independent experiments. *, *p* < 0.05; **, *p* < 0.01.

To further evaluate whether caveolae are involved in ARV endocytosis, Vero and DF-1 cells were pretreated with various concentrations of nystatin (10, 20, and 30 μM). As shown in Figure 6A,B, nystatin caused a significant reduction in μNS and σC mRNA levels, and the inhibitory effect was enhanced with increasing drug concentrations. In addition, the protein levels of the μNS gene (Figure 6C,D) and syncytium formation (Figure 6E,F) also significantly inhibited by nystatin, in a dose-dependent manner. The data indicated that blocking caveolae-mediated endocytosis reduced the level of ARV infection. Thus, using dynamin, caveolae, and clathrin inhibitors, we found that ARV may enter cells via dynamin-dependent, caveolae-mediated endocytosis and not by clathrin-dependent endocytosis.

## 4. Discussion

Viral entry steps, including virus attachment, penetration, uncoating, and viral genetic program activation, are crucial determinants of virus infection [9]. These steps rely on the viral hijacking of normal cellular progress. Although it is believed that ARV bind to the cell receptor via the σC fiber and are internalized into cells by endocytosis [5,37], the mechanism of the host endocytic pathway utilized by ARV entry remains elusive. Caveolae is a specialized form of lipid raft that plays an essential role in endocytic viral entry, and Cav-1 is the principal structural protein of caveolae [15,16]. Our previous study suggested that cellular cholesterol in lipid rafts plays a critical role in ARV replication, and ARV σC fibers do not co-localize with Cav-1 when virus particle attachment proceeds without endocytosis [22]. Therefore, it is likely that ARV particle-receptor complexes move and localize to caveolae after virions bind to non-raft lipid areas within the membrane, followed by caveolae-dependent endocytosis. However, evidence of the direct involvement of caveolae in ARV entry is still lacking, and whether Cav-1 interacts with ARV viral protein during endocytosis needs to be characterized.

ARV σB, the major outer capsid protein, which interacts with μB to form the outer capsid, appears to be responsible for early virus–host interactions in ARV infection and produces group-specific antibody against ARV [4,27]. Although there are a few reports on the development of ARV vaccines and diagnostic reagents based on σB [38], there is no report on the properties and activities of this protein or its role in viral pathogenesis. In this study, ARV capsid protein σB was found to associate with Cav-1 in DRMs fractions, suggesting that the ARV σB is probably associated with caveolar lipid rafts at the ARV entry stage. Furthermore, the σB protein co-localized and immunoprecipitated with Cav-1. Thus, we demonstrated for the first time the interaction between the ARV σB protein and Cav-1 during the virus entry stage.

With reference to the research on reovirus, the outer capsid is composed of a heterohexameric complex of σ3 protein (a counterpart of ARV σB) and μ1 protein (a counterpart of ARV μB). Within the endocytic compartment, reovirus σ3 acts as a “protector” protein for endosomal membrane-penetration protein μ1. Therefore, the outer-capsid protein σ3 should be removed by cathepsin proteases prior to the exposure of the hydrophobic conformer of the μ1 protein [39,40]. In the present study, the σB–Cav-1 interaction confirmed the involvement of caveolae in ARV entry in this study. Based on the information on reovirus σ3 described above, we proposed that the σB–Cav-1 interaction probably occurred in cell-surface caveolae and caveosomes during endocytosis, before the proteolytic degradation of σB. Nevertheless, the detailed mechanism of the interaction between σB and Cav-1 needs to be further studied.

Specific inhibitory drugs are the most common method used to evaluate the viral entry pathway [15]. In the present study, to further validate the important role of caveolae in ARV endocytosis and clarify the detail entry routes of ARV, the significance of dynamin, caveolae, and clathrin during ARV entry were investigated by specific chemical inhibitors, both in Vero and DF-1 cells. The results here revealed that dynasore and nystatin treatment both remarkably inhibited ARV entry in a dose-dependent manner, while CPZ treatment had little influence on ARV entry. This implies that ARV enter Vero and DF-1 cells through a caveolae-mediated endocytosis pathway that depends on dynamin, while clathrin is not required for the entry. The data were consistent with the results mentioned that the ARV σB protein is associated with Cav-1.

In conclusion, our data confirmed that ARV σB protein interacts with Cav-1 during dynamin-dependent, caveolae-mediated endocytosis for virus entry. This is the first study to demonstrate the interaction between the ARV σB protein and Cav-1 during the virus entry stage. The current study augments our understanding of the virus–host interactions during the ARV internalization and the entry routes of ARV. It may help to pave the way and provide new insights into the pathogenesis and control of ARV.

## Figures and Tables

**Table 1:** The primer sequences for qRT-PCR.

Primer Name	Primer Sequence (5′-3′)
σC-F	CGTATCATTCACCCGCGATT
σC-R	TGTTCGCTGTACCATCACCT
μNS-F	CGTGTGGAAGCGTTAAACCA
μNS-R	TCATCACGCTCGTTCAGGTA
β-actin-F (chicken)	ATTGTCCACCGCAAATGCTTC
β-actin-R (chicken)	AAATAAAGCCATGCCAATCTCGTC
β-actin-F (monkey)	GGCCAGGTCATCACCATT
β-actin-R (monkey)	ATGTCCACGTCACACTTCATG

## Data Availability

Not applicable.
